# Vocal and auditory symptoms and self-perception of voice and hearing in voice actors

**DOI:** 10.1590/2317-1782/e20240316en

**Published:** 2025-12-05

**Authors:** Heloísa Soares Povreslo, Ana Carolina Constantini

**Affiliations:** 1 Departamento de Desenvolvimento Humano e Reabilitação, Universidade Estadual de Campinas – Unicamp - Campinas (SP), Brasil.

**Keywords:** Worker Health, Voice, Voice Disorders, Hearing, Hearing Loss

## Abstract

**Purpose:**

To trace suspected voice disorder based on vocal and auditory symptoms self-reported by voice actors and analyze the correlation between these symptoms.

**Methods:**

Quantitative, cross-sectional and prospective study, approved by the institution’s Research Ethics Committee. An online questionnaire was applied consisting of different instruments to trace vocal and auditory symptoms and conditions: Vocal Production of the Actor (VP-A), Screening Index for Voice Disorder (SIVD) and Self-Reported Hearing Loss Questionnaire (SRHLQ). Descriptive and inferential statistical analysis was conducted with the application of Fisher’s Exact test to verify the association between suspected voice disorder (VD) and perceived hearing loss (HL) and variables of interest; and the Mantel-Haenszel test (p<0.05) was applied to measure the association between two variables controlled by a third variable.

**Results:**

55 voice actors (27 men, 26 women and 1 non-binary person) composed the sample. An association between suspected VD with work habits, setting and organization and use of vocal technique was noted; self-perception of hearing and perception of HL was associated with habits and perceptions about the voice. Seven voice actors (12.7%) presented suspected VD and 31 (56.4%) presented perceived HL. The most common vocal symptom was throat clearing. Most of those with perceived HL (14, 25.5%) were aged up to 35 years.

**Conclusion:**

Self-perceived HL affected most participants and suspected VD was less frequent. There was an association between suspected VD and vocal symptoms and also with hearing.

## INTRODUCTION

Voice actors are part of a professional category that uses the voice to perform their work functions. Voice actors are actors who use the voice to adapt audiovisual productions that are originally spoken in a foreign language by dubbing it in a country’s mother tongue. These professionals have also worked in providing voice for video game characters, and several of these games, as well as other audiovisual productions, involve high vocal demand for the production of screams, among other extreme vocal productions^(^1,2^)^.

These professionals are expected to have the following vocal characteristics: flexibility to be able to give life to different characters with different profiles (including cartoons); and resistance to endure prolonged working hours^(^2-4^)^ in high vocal demand, with the need for extreme vocal productions, such as screams.

In addition to the characteristics of voice use, it is important to consider the environmental and organizational characteristics of the work^(^1,5^)^ of these professionals, such as the performance of different simultaneous activities that require vocal use^(^3,4^)^. A previous study analyzing voice professionals showed that the voice actor group exhibited more phonotraumas than other professional groups^(^6^)^, which may suggest a vocal risk resulting from this professional activity.

Voice actors need to produce character voice and simultaneously listen to audio of the production in the original language, which requires important auditory feedback to obtain perfect synchrony between actor movement in the original production and in that which will have the voice inserted^(^7^)^. There is a known intrinsic relation between vocal production and auditory system; therefore, hearing difficulties can directly impact vocal production^(^8,9^)^. However, despite this relation being established in voice actors, studies on this relation are scarce^(^10^)^.

Despite being a consolidated profession in several countries, studies on voice actors’ vocal production are fewer compared to studies with other categories of voice professionals. The increasing number of streaming services worldwide should increasingly require the work of these professionals in dubbing speech in audiovisual productions translated into the mother tongue of each country^(^11^)^. In the Brazilian context, the consumption of dubbed productions may be related to socio-educational issues, such as the level of literacy of the Brazilian population, which makes dubbers quite in demand.

Knowing the risk of developing vocal disorders, and the vocal and auditory symptoms self-perceived by this population, is important to assist in planning vocal advisory and preventing dysphonia, in addition to favoring a more complete assessment of this professional. This study aims to trace suspected voice disorder based on vocal and auditory symptoms self-reported by voice actors and analyze the correlation between these symptoms.

## METHOD

This is a study with a quantitative and cross-sectional, prospective approach, approved by the institution’s ethics committee under opinion No. 5,967,931.

### Instruments

An instrument containing three questionnaires validated in Brazilian Portuguese was built and made available on a free online platform (Google Forms).

Since the voice actors are also actors, the Vocal Production of the Actor – VP-A^(^12^)^ instrument was used, consisting of 56 questions. The answers to the questions are categorized into a likert scale, with the following possibilities: 0 (never), 1 (rarely), 2 (sometimes), 3 (almost always) and 4 (always). The VP-A is subdivided into five domains, namely: *identification* (sociodemographic information – date of birth, gender, marital status and education), *functional status* (with questions about time working in the profession and type of training, for example), *work setting* (with questions about the participant’s main work setting), *work organization* (about relationship with the team, for example) and *vocal aspects, habits and lifestyle* (with questions about alcohol and energy drink intake, water consumption, vocal warm-up and cool-down, absences from work due to voice disorders, among others). As it is a questionnaire available to actors, 10 questions and the guidelines on questions 12 to 25 were adapted considering the specificities of voice actors, such as using the term “voice actor” instead of “actor.” Chart 1 shows the modifications.

**Chart 1:** Comparison between the questions available in VP-A and those made available in the form after reformulation

**VP-A question**	**Question available on the form**
6. How long have you been a professional actor?	6. How long have you been a professional actor/voice actor?
7. What is your type of actor training?	7. What is your type of training in this profession?
8. How many play, projects and other professional activities are you currently involved in? (consider the last 30 days for answering)	8. How many dubbing projects are you currently involved in? (consider the last 30 days for answering)
8.1. How many hours per week do you dedicate to performing and rehearsing?	8.1. How many hours per week do you dedicate to recording and rehearsing?
9. In addition to acting, do you perform other activities that require the use of voice?	9. In addition to voice acting, do you perform other activities that require the use of voice?
9.1. Can you support yourself financially with your profession as an actor?	9.1. Can you support yourself financially with your profession as a voice actor?
11. What function(s) do you currently perform in the theatre (please answer for each function type).	11. What function(s) do you currently perform in the voice acting studio (answer for each function type).
13. Is your rehearsal and performance space noisy?	13. Is your rehearsal and recording space noisy?
26.1. Do you have a good relationship with your colleagues who work in the play (other actors, production and technical staff)?	26.1. Do you have a good relationship with your colleagues who work in the recording (other actors, production and technical staff)?
39.6. Which of the situations of violence listed below have already occurred in your work setting: Violence around the rehearsal/performance setting.	39.6. Which of the situations of violence listed below have already occurred in your work setting: Violence around the rehearsal/recording setting.
Guidance on questions 12–25 about the work setting: “Think about the places where you usually rehearse and perform. The questions below should be answered considering your main work setting.”	Guidance on questions 12–25 about the work setting: “Think about the places where you usually rehearse and record. The questions below should be answered considering your main work setting.”

Source: Prepared by the authors, 2025

The Screening Index for Voice Disorder – SIVD^(^13^)^, with 12 questions related to the tracing of vocal symptoms, was also used. It has the possibility of answering with “never,” “rarely,” “sometimes,” and “always,” and scores above or equal to 5 are considered suggestive of voice disorder (answers with *sometimes* or *always* have a score equal to 1; other frequencies are not scored). The SIVD score indicates suspected voice disorder and suggests the need for further evaluation.

The third questionnaire applied was the Self-Reported Hearing Loss Questionnaire^(^14^)^, hereinafter referred to as SRHLQ, which has 3 questions. positive cases of self-reported hearing loss were considered when there was at least one of the following answers: “yes” to question “1- Do you feel you have hearing loss; “regular” and “poor” to “2- In general, would you say that your hearing is:”; and any answer except “You hear the same way you heard before” to “3- Currently, do you think that:”.

### Subjects

The study’s call for participation was published on social networks and in contact with dubbing studios, and the non-probabilistic sample was constituted by convenience. The inclusion criteria were: being a professional voice actor, of different genders, aged over 18 years, without restriction regarding the time working in the profession, who agreed to participate in the study by signing the Informed Consent Form. The exclusion criteria were: self-reported history (current or previous) of cancer in the larynx region or underlying neurological disease. The sample consisted of 55 voice actors.

### Data analysis

For statistical analysis, the Python (version 3.12)^(^15^)^ and R (version 4.3.1)^(^16^)^ programming languages were used. For inferential statistical analysis, the following tests were applied: Fisher’s Exact test (p-value <0.05) was used to verify whether two variables are independently related or whether there is any significant association between them; the Mantel-Haenszel test was used to measure the association between two variables controlled by a third variable; and multiple correspondence analysis was used to identify a group of individuals with similar profiles. Descriptive statistical analysis used mean values, absolute frequency and percentage of sociodemographic variables, in addition to the total SIVD score and the presence of at least one positive answer in the SRHLQ.

To test the associations, the variables of interest (gender, age, education, time since training in the profession, type of training, performance of other activities with vocal demand, smoking, consumption of alcoholic and energy drinks, water intake during voice use, absences from work due to voice issues, performance of vocal warm-up and cool-down) were used by cross-relating them to the SIVD answers (each vocal symptom and positive or negative score for VD) and the SRHLQ answers.

## RESULTS

Research participants included 55 people, of which 27 males, 26 females and 1 trans/non-binary person, with a mean age of 40 years (min = 26; max = 74). As for training, 21 (38.2%) reported stage training, higher education or vocational technical course and workshops; 12 (21.8%) reported stage training and higher education/vocational technical course; 6 (10.9%) reported stage training; 6 (10.9%) reported stage training and workshops; 5 (9.1%) reported higher education/vocational technical course and workshops; and 5 (9.1%) reported higher education or vocational technical course. In the sample studied, 23 individuals (42.6%) reported time working as a voice actor below or equal to 5 years. Smoking was mentioned by 12 subjects (21.8%). Voice use for other activities was reported by 44 participants (84.6%). Sample characterization as to variables for age and time working in the profession are shown in Table 1.

**Table 1:** Characterization of study sample regard to age and years of professional experience

		n	%
Age (n=55)	Between 36 and 40	15	27.3
	Between 31 and 35	12	21.8
	Over 50	11	20.0
	Under 30	9	16.4
	Between 41 and 50	8	14.5
Years of professional experience (n=54)	Less than 5	23	42.6
	Between 5 and 10	16	29.6
	More than 10	15	27.8

Caption: n = number of participants who responded to the question

Variables of interest were selected by the study researchers in the VP-A instrument and these variables had their associations tested by Fisher’s Exact test (p<0.05) with the presence or absence of suspected voice disorder according to the SIVD and the absence or presence of perceived hearing loss according to the SRHLQ. Table 2 shows the pairs of variables that were associated with each other, with emphasis on the associations between positive SIVD variables (suspected voice disorder) with habits and food, work setting and organization, use of vocal technique, investigated through VP-A, and self-perception of hearing and perception of hearing loss with association with habits and perceptions about voice.

**Table 2:** Variable pairs of interest with statistically demonstrated association by the Exact test Fisher’s

Pairs	p-value
**Gender**	
Gender × Absence due to voice disorder	0.001
Gender × Voice loss	0.022
**Years of professional experience × Strained speech**	0.001
**Habits**	
Consumption of alcoholic drink × Low-pitched voice	0.040
Consumption of energy drink × Vocal cool-down	0.026
Consumption of energy drink × Hoarseness	0.031
Consumption of energy drink × Cough with secretion	0.019
Consumption of energy drink × Secretion/phlegm in throat	0.014
Consumption of energy drink × Strained speech	0.005
Water intake × Vocal warm-up	0.007
Water intake × Breaking voice	0.043
Vocal warm-up × Use of vocal technique	0.008
Vocal cool-down × Hoarseness	0.042
**Absence due to voice disorder × Strained speech**	0.028
**Positive SIVD**	
SIVD × Hoarseness	0.013
SIVD × Low-pitched voice	0.044
SIVD × Vocal cool-down	0.036
SIVD × Dry cough	0.010
SIVD × Cough with secretion	0.000
SIVD × Secretion/phlegm in throat	0.000
SIVD × Dry throat	0.003
SIVD × Strained speech	0.000
SIVD × Hearing	0.045
**Hearing Self-Assessment**	
Hearing Self-Assessment × Consumption of energy drinks	0.004
Hearing Self-Assessment × Low-pitched voice	0.014
**Self-perception of hearing loss**	
Self-perception of hearing loss × Consumption of energy drink	0.045
Self-perception of hearing loss × Water intake	0.037
Self-perception of hearing loss × Hearing	0.003

**Figure 1:**
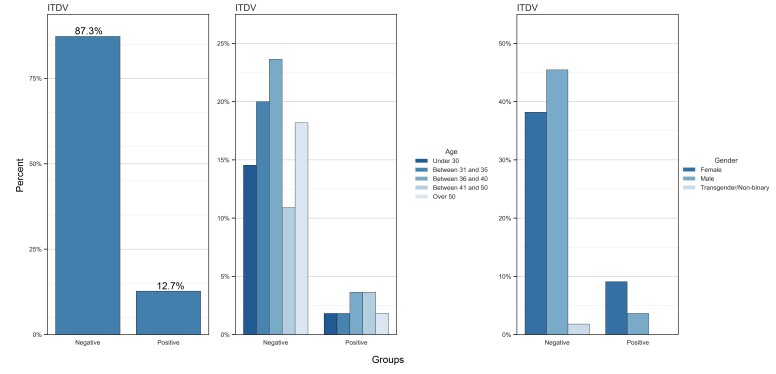
Distribution of subjects in the sample according to the SIVD score, age and gender

Figure 1 shows the distribution of SIVD results, being positive (n=7) the cases in which the subjects exhibit suspected voice disorder, and shows the relation between suspected voice disorder according to participant age and gender. Most study participants (with both positive and negative SIVD) are in the period of maximum vocal efficiency (n= 42, 76.4%). Among those with suspected voice disorder, most are women who are in the 36–40 age range.

In the SRHLQ, 31 (56.4%) participants had positive answers for self-reported hearing loss. In this instrument, 30.9% (n=17) of the participants had a positive perception of hearing loss, 5.5% (n=3) reported feeling regular or poor hearing, and 51.9% (n=28) perceived changes in auditory perception (currently believe they heard better before).

Among the participants who have the perception of hearing loss, most are aged up to 35 years. In addition, male participants had a higher perception of hearing loss and more positive responses to self-reported hearing loss.

Voice actors are a recognized category of voice professionals with unique vocal demand. Regarding the distribution of responses that relate suspected voice disorder (screened by SIVD) and self-perceived hearing loss reported by the participants, the majority (n=26, 47.3%) presented only self-perceived hearing loss. Only 7 subjects (12.7%) had scores suggestive of VD, which may or may not be associated with self-perceived hearing loss, and 9.1% (n=5) had an association of both. Other participants (n=22, 40%) did not present suspected voice disorder and/or self-perceived hearing loss.

To test a possible association between positive SIVD and positive hearing loss, the variables of these instruments were fixed via statistical analysis with Mantel-Haenszel test and only the presence of throat clearing showed significance (p-value = 0.031). However, the study sample was small and some associations between the frequency of throat clearing and positive/negative hearing loss did not present any subject with the response, making this test little robust. Considering the positive SIVD, there were no participants who reported clearing their throat *never* or *rarely*, regardless of the scores for self-reported hearing loss; in addition, for both positive SIVD and negative SIVD, there were no subjects who reported *always* presenting this symptom and also presented scores suggestive of self-reported hearing loss.

## DISCUSSION

Voice actors constitute a public of potential interest for the field of Speech-Language Pathology both in relation to voice (since they are voice professionals and have unique vocal demands) and to hearing, since it is important for the regulation of vocal production, with auditory feedback being essential for vocal self-monitoring^(^8^)^. Studies that analyze this relation in voice actors are scarce. Therefore, our study innovates by seeking to trace suspected voice disorder, vocal symptoms and auditory symptoms reported by professional voice actors and correlate these symptoms.

Regarding age, most (n=27, 49.1%) of the participants were aged between 31 and 40 years, a period of maximum vocal efficiency (between 25 and 45 years of age)^(^17^)^. Most voice actors (n=23, 42.6%) had time working in the profession below or equal to 5 years.

Considering the voice actors’ vocal demand context, most participants were expected to have suspected voice disorder during SIVD screening, which was not observed. A hypothesis for this finding may be related to previous training for professional vocal skills for the dubbing activity, since 54 participants reported having received previous vocal advisory. Such advisory had been mostly obtained from other actors/directors, speech-language pathologists and/or singing teachers (n=16, 29.6%). In addition, 80% of the individuals (n=44) reported using their voice in other activities (including narration, voiceover, singing, original voice, acting in theater, singing and/or theater classes – teacher and student), which can also contribute to previous training of professional vocal skills, ensuring lower impact on vocal health and consequently less suspected voice disorders.

On the other hand, a higher proportion of auditory complaints was observed, which can impact vocal performance. Since auditory feedback is fundamental for speech production^(^18^)^, subjects who present more auditory complaints could exhibit more vocal complaints. It can be assumed that even participants who did not present suspected voice disorder may present it in the future.

Studies on the hearing of voice actors relate only to Central Auditory Processing. A previous study^(^19^)^ demonstrated that voice actors do not exhibit auditory effort, a term whose definition still lacks consensus, but which is commonly presented as the attention and cognitive effort required to understand a spoken message^(^20^)^. However, this population presented central auditory processing disorder, with greater impairment in auditory figure-ground skills^(^19^)^ (related to listening in noisy environments^(^21^)^) and binaural interaction^(^19^)^ (which refers to the processing of complementary auditory information from both ears simultaneously).

It is noted that voice actors routinely use headphones in their work routine, which could cause hearing complaints, with potential to cause irreversible hearing losses in this population, depending on the intensity of and time of exposure to noise^(^22-24^)^. The frequent use of these devices has been associated with bilateral sensorineural hearing loss^(^22^)^. The use of headphones could also justify the responses of the participants of the present study, who mostly (n=31, 56.4%) presented positive responses for perceived hearing loss in the SRHLQ. Eleven participants (20.4%) reported decreased hearing in both ears. In addition, four participants (7.4%) reported that only the right ear started to hear less, while three voice actors (5.6%) noticed this reduction only in the left ear. It is believed that inadequate practices, such as constant use of headphones unilaterally, without adequate exchange between the ears, can contribute to these perceptions. However, the instrument answered by the participants did not include questions about the headset. It should be noted that the SRHLQ analyzes the perception of the participants, which does not necessarily reflect the diagnosis of hearing loss. Thus, there is evident need for more in-depth clinical work and scientific research on this subject to study whether the perception of hearing loss is equivalent to the diagnosis, for example.

As shown in Table 2, several associations were found. It is observed that some habits traced by the VP-A (such as consumption of energy drinks and water intake) showed relation with voice self-assessment by voice actors. The intake of energy drinks is particularly relevant in the sample studied, as it was also associated with the habit of performing vocal cool-down and five symptoms of the SIVD (thick voice, hoarseness, cough with secretion, secretion in the throat and tiredness when speaking). In addition, this habit was also associated with poor self-assessment of hearing (p-value 0.004) and self-perception of hearing loss (p-value 0.045). Caffeine, present in high dosages in energy drinks, acts on the Central Nervous System (CNS), impacting cognition in order to improve attention time^(^25-28^)^ and impacts the productivity of individuals. One hypothesis for this finding could be related to the long working hours that the participants indicated (reaching 60 hours per week), culminating in the use of these beverages to overcome fatigue and meet the exhaustive working hours.

It is noted that the SIVD score was associated with several vocal symptoms that are present within the VP-A, which may suggest a convergence between the participants’ responses for such symptoms and the relevance of applying this instrument in this population.

Among the research participants, 41.8% (n=23) *rarely* or *never* manage to support themselves financially and 80% (n=44) report performing other activities with the use of voice, often in other professions (teacher, theater actor, singer, audiobook and/or original voice recording, voiceover, dubbing direction, among others). One hypothesis for this finding is that these characteristics in the participants favor higher vocal and auditory risks, since it is assumed that the context to which they are subject leads to the need for more working hours, which would also justify the consumption of energy drinks mentioned above.

Studies on vocal symptoms in voice professionals are common; however, specific studies focused on voice actors are rarer. A previous study^(^29^)^ showed that the most relevant symptoms for voice actors after a recording were: throat dryness, constant throat clearing, shoulder and neck strain, and hoarseness. In our study, despite the use of different instruments from the previously mentioned study, these vocal symptoms were also reported by the participants, but only throat clearing showed significant responses, being reported by most voice actors in the frequencies *sometimes* and *always*. Throat clearing has also been reported in a study with other populations using the VP-T, as in the comparison between evangelical pastors and the control group^(^30^)^. Finally, a relation was obtained between suspected VD and gender, being higher in women. This finding may be related to the anatomical conditions presented by *cisgender* women, which are favorable for the development of vocal issues^(^31-34^)^.

As in all scientific studies, this one also has some limitations, since there is no validated instrument for voice actors and, therefore, the VP-A was used with modifications in some questions to increase the specificity for the study population; although voice actors are also actors (which justifies the use of the instrument), they are subject to specific demands during their work in voice acting. The SIVD is a validated instrument built for screening voice disorders in teachers; however, the questions refer to vocal symptoms commonly experienced by people with voice disorders and that are not necessarily related to teaching. Therefore, it can be perfectly fit into the sample composed of actors. It is important to note that this study only addressed the self-perception of these subjects about vocal and auditory symptoms. Despite representing an important item of the multidimensional assessment of voice, it is not enough for a detailed assessment of voice in these professionals. In future studies, it is important to research auditory-perceptual judgment and acoustic aspects, in addition to audiological tests to confirm or refute hearing loss in this population. However, the findings demonstrate the need for a broad approach in the speech-language pathological assessment of voice actors, emphasizing the auditory and vocal aspects, in order to promote the health of these workers.

## CONCLUSION

The most frequent vocal symptom for the participants was throat clearing, followed by hoarseness. Suspected voice disorder affected fewer participants than self-perceived hearing loss. There was an association between the SIVD score and vocal symptoms and also with hearing. Only the throat clearing symptom showed a significant association with the SIVD and SRHLQ. Considering this symptom, participants who had negative SIVD reported less self-reported hearing loss than voice actors with positive SIVD.
