# Supplementary material for: Vocal and auditory symptoms and self-perception of voice and hearing in voice actors
Source: Codas. 2025 Dec 5;37(6):e20240316. doi: 10.1590/2317-1782/e20240316en (PMC12704606; doi:10.1590/2317-1782/e20240316en)
Supplement: Translation

# Translation: Sintomas vocais, auditivos e autopercepção de voz e audição em dubladores

**DOI:** 10.1590/2317-1782/e20240316pt

**Authors:** Heloísa Soares Povreslo, Ana Carolina Constantini

**Affiliations:** 1 Departamento de Desenvolvimento Humano e Reabilitação, Universidade Estadual de Campinas – Unicamp - Campinas (SP), Brasil.

**Keywords:** Saúde do Trabalhador, Voz, Distúrbios da Voz, Audição, Perda Auditiva

## Abstract

**Objetivo:**

Identificar a suspeita de distúrbio de voz com base em sintomas vocais e auditivos autorreferidos por dubladores e analisar a correlação entre esses sintomas.

**Método:**

Estudo quantitativo, corte transversal e prospectivo, aprovado pelo Comitê de Ética em Pesquisa da instituição. Aplicou-se virtualmente questionário composto por diferentes instrumentos para identificação dos sintomas e condições vocais e auditivas: Condição de Produção Vocal do Ator (CPV-A), Índice de Triagem para Distúrbio de Voz (ITDV) e Questionário de perda auditiva autorreferida (QPAAR). Realizou-se análise estatística descritiva e inferencial, com a aplicação do teste Exato de Fisher para verificar associação entre suspeita de distúrbio de voz (DV) e percepção de perda auditiva (PA) e variáveis de interesse; e do teste de Mantel-Haenszel (p<0,05) para medir a associação entre duas variáveis controlado por uma terceira variável.

**Resultados:**

55 dubladores (27 homens, 26 mulheres e 1 pessoa não-binária) compuseram a amostra. Destaca-se associação entre suspeita de DV com hábitos, ambiente e organização de trabalho e uso de técnica vocal; autopercepção da audição e percepção de PA associou-se com hábitos e percepções sobre a voz. Sete dubladores (12,7%) apresentaram suspeita de DV e 31 (56,4%) apresentaram percepção de PA. O sintoma vocal mais comum foi o pigarro. A maioria com percepção de PA (14, 25,5%) tinha idade até 35 anos.

**Conclusão:**

A autopercepção de PA acometeu a maioria dos participantes e a suspeita de DV foi menos frequente. Houve associação entre suspeita de DV e sintomas vocais e também com a audição.

## INTRODUÇÃO

Os dubladores fazem parte de uma categoria profissional que utiliza a voz para o desenvolvimento de suas funções laborais. Dubladores são atores que emprestam suas vozes para a adaptação de produção em audiovisual originalmente em língua estrangeira para substituição da locução na língua materna de um país. Estes profissionais também têm atuado na composição de vozes de personagens de video-games, sendo que muitos desses jogos, assim como outras produções audiovisuais, envolvem alta demanda vocal para produção de gritos, entre outros extremos vocais^(1,2)^.

Como características vocais desses profissionais, espera-se flexibilidade para poderem dar vida a diferentes personagens com perfis diversos (inclusive desenhos animados) além de resistência para o cumprimento de jornadas de trabalho prolongadas^(2-4)^ em alta demanda vocal, com a necessidade de produção de extremos vocais, como os gritos.

Além das características de uso da voz, é importante considerar as características ambientais e organizacionais do trabalho^(1,5)^ destes profissionais, como a realização de diferentes atividades simultâneas que exigem o uso vocal^(3,4)^. Estudo anterior que analisou profissionais da voz mostrou que no grupo em que os dubladores estavam incluídos houve mais presença de fonotraumas do que em outros grupos de profissionais^(6)^, o que pode sugerir um risco vocal decorrente desta atividade profissional.

A necessidade dos dubladores de produzir a voz da personagem e simultaneamente ouvir o áudio da produção na língua original exige importante *feedback* auditivo para dar a perfeita sincronia entre o movimento do ator na produção original e naquela em que a voz será inserida^(7)^. Sabe-se que há intrínseca relação entre produção vocal e sistema auditivo, de forma que dificuldades auditivas podem impactar diretamente na produção da voz^(8,9)^. No entanto, apesar de essa relação ser estabelecida em dubladores, estudos sobre essa relação são escassos^(10)^.

Apesar de ser uma profissão consagrada em diversos países, o estudo da produção vocal de dubladores é menor se comparado a estudos com outras categorias de profissionais da voz. O avanço de serviços de *streamings* de forma mundial deve, cada vez mais, solicitar a atuação destes profissionais na tradução da locução de produções de audiovisual para a língua materna de cada país^(11)^. Na realidade brasileira, o consumo de produções dubladas pode estar relacionado a questões socioeducacionais, como o nível de letramento da população brasileira, o que faz com que dubladores sejam bastante requisitados.

O conhecimento do risco para desenvolvimento de distúrbios vocais, bem como os sintomas vocais e auditivos autopercebidos por essa população, é importante para auxiliar no planejamento de ações de assessoria vocal e prevenção de disfonias, além de favorecer uma avaliação mais completa deste profissional da voz. O objetivo deste estudo foi identificar a suspeita de distúrbio de voz com base em sintomas vocais e auditivos autorreferidos por dubladores e analisar a correlação entre esses sintomas.

## MÉTODO

Estudo com abordagem quantitativa e de corte transversal, prospectivo, aprovado pelo comitê de ética da instituição sob parecer nº 5.967.931.

### Instrumentos

Um instrumento contendo três questionários validados no português brasileiro foi construído e disponibilizado em plataforma virtual gratuita (*Google Forms*).

Visto que os dubladores também são atores, utilizou-se o instrumento Condição de Produção Vocal do Ator – CPV-A^(12)^, composto por 56 questões. O registro das respostas às perguntas é dado em escala likert, com as possibilidades: 0 (nunca), 1 (raramente), 2 (às vezes), 3 (quase sempre) e 4 (sempre). O CPV-A é subdividido em cinco domínios, sendo estes: *identificação* (informações sociodemográficas – data de nascimento, gênero, estado civil e escolaridade), *situação funcional* (com perguntas sobre tempo de atuação na profissão e tipo de formação, por exemplo), *ambiente de trabalho* (com perguntas sobre o ambiente principal de trabalho do participante), *organização do trabalho* (sobre relacionamento com a equipe, por exemplo) e *aspectos vocais, hábitos e estilo de vida* (com perguntas sobre ingestão de bebidas alcoólicas e energético, consumo de água, realização de aquecimento e desaquecimento vocal, faltas no trabalho por alteração vocal, entre outros). Por se tratar de um questionário disponível para atores, 10 questões e as orientações sobre as questões de 12 a 25 foram adaptadas considerando as especificidades dos dubladores, como utilizar o termo “dublador” ao invés de “ator”. O Quadro 1 apresenta as modificações realizadas.

**Quadro 1:** Comparação entre as perguntas disponíveis no CPV-A e as disponibilizadas no formulário após reformulação

**Pergunta CPV-A**	**Pergunta disponível no formulário**
6. Há quanto tempo você é ator profissional?	6. Há quanto tempo você é ator/dublador profissional?
7. Qual o seu tipo de formação de ator?	7. Qual o seu tipo de formação nesta profissão?
8. Em quantos peças, projetos e demais atividades profissionais você está envolvido atualmente? (considere os últimos 30 dias para a resposta)	8. Em quantos projetos de dublagem você está envolvido atualmente? (considere os últimos 30 dias para a resposta)
8.1.Quantas horas por semana você dedica para se apresentar e ensaiar?	8.1.Quantas horas por semana você dedica para realizar gravações e ensaiar?
9. Além de atuar, você realiza outras atividades que exigem o uso da voz?	9. Além de dublador, você realiza outras atividades que exigem o uso da voz?
9.1. Você consegue se manter financeiramente com a sua profissão de ator?	9.1. Você consegue se manter financeiramente com a sua profissão de dublador?
11. Qual(is) função(ões) você desempenha atualmente no teatro (responda para cada tipo de função).	11. Qual(is) função(ões) você desempenha atualmente no estúdio de dublagem (responda para cada tipo de função).
13. Seu espaço de ensaio e apresentação é ruidoso?	13. Seu espaço de ensaio e gravação é ruidoso?
26.1.Você tem bom relacionamento com seus colegas que trabalham com a peça (demais atores, produção e equipe técnica)	26.1.Você tem bom relacionamento com seus colegas que trabalham na gravação (demais atores, produção e equipe técnica).
39.6. Quais das situações de violência relacionadas abaixo já ocorreram no seu ambiente de trabalho: Violência ao redor do ambiente de ensaio/apresentação.	39.6. Quais das situações de violência relacionadas abaixo já ocorreram no seu ambiente de trabalho: Violência ao redor do ambiente de ensaio/gravação.
Orientações sobre as questões de 12 a 25 sobre o ambiente de trabalho: “Pense nos locais em que costuma ensaiar e se apresentar. As questões abaixo deverão ser respondidas pensando no seu ambiente principal de trabalho”.	Orientações sobre as questões de 12 a 25 sobre o ambiente de trabalho: “Pense nos locais em que costuma ensaiar e fazer gravações. As questões abaixo deverão ser respondidas pensando no seu ambiente principal de trabalho”.

Fonte: Elaborado pelas autoras, 2025

Também foi utilizado o Índice de Triagem para Distúrbio de Voz – ITDV^(13)^, com 12 questões relacionadas à identificação de sintomas vocais. Apresenta possibilidade de respostas “nunca”, “raramente”, “às vezes” e “sempre”, no qual são considerados casos sugestivos de distúrbio de voz os escores maiores ou iguais a 5 (respostas *às vezes* ou *sempre* apresentam pontuação igual a 1; demais frequências não são pontuadas). O escore do ITDV indica a suspeita de distúrbio de voz e sugere necessidade de avaliação mais detalhada.

O terceiro questionário aplicado foi o Questionário de perda auditiva autorreferida^(14)^, doravante denominado QPAAR, que apresenta 3 questões. São considerados casos positivos de perda auditiva autorreferida ao menos uma das seguintes respostas: “sim” para a pergunta “1- Você sente que tem perda auditiva; “regular” e “ruim” para “2- Em geral, você diria que sua audição é:”; e qualquer resposta exceto “ouve da mesma forma que ouvia antes” para “3- Atualmente, você acha que:”.

### Sujeitos

A chamada para participação no estudo foi divulgada em redes sociais e em contato com estúdios de dublagem, sendo a amostra não-probabilística constituída por conveniência. Os critérios de inclusão foram dubladores profissionais, de gêneros diversos, maiores de 18 anos, sem restrição com relação ao tempo de atuação na profissão, que aceitassem participar do estudo assinando o Termo de Consentimento Livre e Esclarecido. Os critérios de exclusão foram histórico autorreferido (atual ou pregresso) de câncer na região da laringe ou doença neurológica de base. A amostra foi composta por 55 dubladores.

### Análise dos dados

Para a análise estatística, foram utilizadas as linguagens de programação Python (versão 3.12)^(15)^ e R (versão 4.3.1)^(16)^. Com relação à análise estatística inferencial, foram aplicados os seguintes testes: teste Exato de Fisher (p-valor <0,05), utilizado para verificar se duas variáveis estão relacionadas de forma independente ou se existe alguma associação significativa entre elas; o teste de Mantel-Haenszel, para medir a associação entre duas variáveis controlado por uma terceira variável; e análise de correspondência múltipla, para identificar um grupo de indivíduos com perfis semelhantes. A análise estatística descritiva utilizou valores de média, frequência absoluta e porcentagem das variáveis sociodemográficas, além do escore total do ITDV e a presença de ao menos uma resposta positiva no QPAAR.

Para testar as associações foram utilizadas as variáveis de interesse (gênero, idade, escolaridade, tempo de formação na profissão, tipo de formação, realização de outras atividades com demanda vocal, fumo, consumo de bebidas alcoólicas e energéticos, ingestão de água durante o uso da voz, faltas no trabalho por alteração na voz, realização de aquecimento e desaquecimento vocal) cruzadas com as respostas do ITDV (cada sintoma vocal e escore positivo ou negativo para DV) e do QPAAR.

## RESULTADOS

Participaram da pesquisa 55 pessoas, sendo 27 do gênero masculino, 26 do gênero feminino e 1 pessoa trans/não-binária, com média de 40 anos de idade (min = 26; máx = 74). Em relação à formação, 38,2% (n=21) referiram formação de palco, graduação ou curso técnico profissionalizante e workshops; 12 (21,8%) apresentam formação de palco e graduação/curso técnico profissionalizante; 6 (10,9%) possuem formação de palco; 6 (10,9%) têm formação de palco e workshops; 5 (9,1%) mencionaram apresentar graduação/curso técnico profissionalizante e workshops; e 5 (9,1%) referiram graduação ou curso técnico profissionalizante. Na amostra estudada, 23 indivíduos (42,6%) referem tempo de atuação como dublador menor ou igual a 5 anos. O hábito de fumar foi mencionado por 12 sujeitos (21,8%). O uso da voz para outras atividades foi referido por 44 participantes (84,6%). A caracterização da amostra com relação às variáveis idade e tempo de atuação estão evidenciadas na Tabela 1.

**Tabela 1:** Caracterização da amostra do estudo com relação à idade e tempo de atuação

		n	%
Idade (n=55)	Entre 36 e 40	15	27,3
	Entre 31 e 35	12	21,8
	Mais que 50	11	20,0
	30 ou menos	9	16,4
	Entre 41 e 50	8	14,5
Tempo de atuação (n=54)	5 ou menos	23	42,6
	Entre 5 e 10	16	29,6
	Mais que 10	15	27,8

Legenda: n = número de participantes que responderam à questão

Foram selecionadas variáveis de interesse pelas pesquisadoras do estudo no instrumento CPV-A e estas variáveis tiveram suas associações testadas via teste Exato de Fisher (p<0,05) com a presença ou ausência de suspeita de distúrbio de voz pelo ITDV e pela ausência ou presença de percepção de perda auditiva via QPAAR. A Tabela 2 demonstra os pares de variáveis que apresentaram associação entre si, com destaque para as associações entre variáveis do ITDV positivo (suspeita de distúrbio de voz) com hábitos e alimentação, ambiente e organização de trabalho, uso de técnica vocal, investigados por meio do CPV-A, e a autopercepção da audição e percepção de perda auditiva com associação com hábitos e percepções sobre a voz.

**Tabela 2:** Pares de variáveis de interesse com associação demonstrada estatisticamente pelo teste Exato de Fisher

Pares	p-valor
**Gênero**	
Gênero × Falta por alteração na voz	0,001
Gênero × Perda da voz	0,022
**Tempo de atuação** × **Cansaço ao falar**	0,001
**Hábitos**	
Consumo de álcool × Voz grossa	0,040
Consumo de energético × Desaquecimento vocal	0,026
Consumo de energético × Rouquidão	0,031
Consumo de energético × Tosse com secreção	0,019
Consumo de energético × Secreção na garganta	0,014
Consumo de energético × Cansaço ao falar	0,005
Ingestão de água × Aquecimento vocal	0,007
Ingestão de água × Falha na voz	0,043
Aquecimento vocal × Uso de técnica vocal	0,008
Desaquecimento vocal × Rouquidão	0,042
**Falta por alteração na voz** × **Cansaço ao falar**	0,028
**ITDV positivo**	
ITDV × Rouquidão	0,013
ITDV × Voz grossa	0,044
ITDV × Desaquecimento vocal	0,036
ITDV × Tosse seca	0,010
ITDV × Tosse com secreção	0,000
ITDV × Secreção na garganta	0,000
ITDV × Garganta seca	0,003
ITDV × Cansaço ao falar	0,000
ITDV × Audição	0,045
**Autoavaliação da audição**	
Autoavaliação da audição × Consumo de energético	0,004
Autoavaliação da audição × Voz grossa	0,014
**Autopercepção de perda auditiva**	
Autopercepção de perda auditiva × Consumo de energético	0,045
Autopercepção de perda auditiva × Ingestão de água	0,037
Autopercepção de perda auditiva × Audição	0,003

A Figura 1 apresenta a distribuição dos resultados do ITDV, sendo positivo (n=7) os casos em que os sujeitos apresentam suspeita de distúrbio de voz, e apresenta a relação entre a suspeita de distúrbio de voz de acordo com a idade dos participantes e o gênero. A maioria dos participantes do estudo (tanto com ITDV positivo, quanto negativo) encontra-se no período de máxima eficiência vocal (n= 42, 76,4%). Dentre aqueles com suspeita de distúrbio de voz, a maioria são mulheres que estão na faixa entre 36 e 40 anos.

**Figura 1:** Distribuição dos sujeitos na amostra segundo o escore do ITDV, a faixa etária e o gênero

No QPAAR, 56,4% (n=31) dos participantes apresentaram respostas positivas para a perda auditiva autorreferida. Neste instrumento, 30,9% (n=17) dos participantes apresentaram percepção positiva de perda auditiva, 5,5% (n=3) referiram sensação de audição regular ou ruim e 51,9% (n=28) perceberam alteração na percepção auditiva (atualmente acreditam que ouviam melhor antes).

Dentre os participantes que têm a percepção de perda auditiva, a maioria tem até 35 anos. Além disso, os participantes do gênero masculino apresentaram maior percepção de perda auditiva, além de mais respostas positivas para a perda auditiva autorreferida.

Os dubladores são uma categoria reconhecida de profissionais da voz e apresentam demanda vocal única. Em relação à distribuição das respostas que relacionam a suspeita de distúrbio de voz (triada pelo ITDV) e autopercepção de perda auditiva relatados pelos participantes, a maioria (n=26, 47,3%) apresentou apenas autopercepção de perda auditiva. Apenas 7 sujeitos (12,7%) apresentaram escores sugestivos de DV, podendo ou não estarem associadas à autopercepção de perda auditiva, sendo que 9,1% (n=5) apresentaram associação de ambas. Demais participantes (n=22, 40%) não apresentaram suspeita de distúrbio de voz e/ou autopercepção de perda auditiva.

Para testar possível associação entre ITDV positivo e perda auditiva positiva, as variáveis destes instrumentos foram fixadas via análise estatística com teste de Mantel-Haenszel e apenas a presença de pigarro demonstrou significância (p-valor = 0,031). Entretanto, a amostra do estudo foi pequena e algumas associações entre a frequência do pigarro e a perda auditiva positiva/negativa não apresentaram nenhum sujeito com a resposta, tornando este teste pouco robusto. Considerando-se o ITDV positivo, não houve participantes que referiram pigarro *nunca* ou *raramente*, independentemente dos escores para perda auditiva autorreferida; além disso, tanto para ITDV positivo quanto ITDV negativo, não houve sujeitos que referiram *sempre* apresentar este sintoma e ainda apresentaram escores sugestivos de perda auditiva autoreferida.

## DISCUSSÃO

Os dubladores são um público de potencial interesse para a Fonoaudiologia tanto em relação à voz (visto que são profissionais da voz e apresentam demandas vocais únicas), quanto à audição, visto que esta é importante para a regulação da produção vocal, sendo o *feedback* auditivo fundamental para o automonitoramento vocal^(8)^. Estudos que analisam essa relação em dubladores são escassos, por isso nosso estudo inova ao buscar identificar a suspeita de distúrbio de voz, os sintomas vocais e auditivos referidos por dubladores profissionais, e correlacionar estes sintomas.

Com relação à idade, a maior parte (n=27, 49,1%) dos participantes apresentou idades entre 31 e 40 anos, período de máxima eficiência vocal (entre os 25 e 45 anos de idade)^(17)^. A maioria dos dubladores (n=23, 42,6%) apresentou tempo de atuação igual ou menor a 5 anos.

Tendo em vista o contexto de demanda vocal dos dubladores, esperava-se que a maioria dos participantes apresentasse suspeita de distúrbio de voz durante a triagem pelo ITDV, o que não foi observado. Uma hipótese para este achado pode estar relacionada com treinamento prévio para as habilidades vocais profissionais para a atividade de dublagem, visto que 54 participantes referiram terem recebido orientações vocais anteriores. Estas orientações foram obtidas, em sua maioria, por outros atores/diretor, fonoaudiólogo e/ou professor de canto (n=16, 29,6%). Além disso, 80% dos indivíduos (n=44) referiram utilizar a voz em outras atividades (dentre as quais foram citados narração, locução, canto, voz original, atuação no teatro, aulas de canto e/ou teatro – professor e aluno), o que também pode contribuir para o treinamento prévio das habilidades vocais profissionais, garantindo menos impacto na saúde vocal e consequente menor suspeita de distúrbio de voz.

Por outro lado, foram observadas em maior proporção queixas auditivas, as quais podem impactar o desempenho vocal. Visto que o *feedback* auditivo é fundamental para a produção vocal^(18)^, sujeitos que apresentam mais queixas auditivas poderiam manifestar mais queixas vocais. Pode-se supor que, mesmo os participantes que não apresentaram suspeita de distúrbio de voz, podem vir a apresentá-la futuramente.

Estudos acerca da audição de dubladores relacionam-se apenas ao Processamento Auditivo Central. Estudo anterior^(19)^ demonstrou que os dubladores não demonstram esforço auditivo, termo para o qual ainda não existe consenso sobre a definição, mas é comumente apresentado como a atenção e o esforço cognitivo necessários para se compreender uma mensagem falada^(20)^. No entanto, esta população apresentou transtorno do processamento auditivo central, com maior prejuízo nas habilidades de figura-fundo^(19)^ (relacionada à escuta em ambientes ruidosos^(21)^) e interação binaural^(19)^ (que se refere ao processamento de informações auditivas complementares advindas das duas orelhas simultaneamente).

Ressalta-se que os dubladores fazem uso rotineiro de fones de ouvido em sua rotina de trabalho, o que poderia ocasionar queixas auditivas, apresentando potencial para gerar perdas auditivas irreversíveis nesta população, a depender da intensidade e tempo de exposição ao ruído^(22-24)^. O uso frequente destes dispositivos já foi associado à perda auditiva do tipo tipo neurossensorial, bilateral^(22)^. O uso dos fones de ouvido também poderia justificar as respostas apresentadas pelos participantes do presente estudo, que, em sua maioria (n=31, 56,4%) apresentaram respostas positivas para percepção de perda auditiva no QPAAR. Onze participantes (20,4%) relataram que a audição em ambos os ouvidos diminuiu em relação ao que era antes. Além disso, quatro participantes (7,4%) afirmaram que apenas o ouvido direito passou a ouvir menos, enquanto três dubladores (5,6%) notaram essa redução apenas no ouvido esquerdo. Acredita-se que más práticas como utilização constante de fone unilateralmente, sem adequada troca entre as orelhas possa contribuir para estas percepções. No entanto, o instrumento respondido pelos participantes não incluía questionamentos sobre o fone de ouvido. É preciso ressaltar que o QPAAR analisa a percepção dos participantes, a qual não necessariamente reflete o diagnóstico de perda auditiva. Desta maneira, fica evidente a necessidade de aprofundamento na atuação clínica e no desenvolvimento de estudos científicos neste tema para investigar se a percepção de perda auditiva se equivale ao diagnóstico, por exemplo.

Assim como demonstrado na Tabela 2, diversas associações foram encontradas. Observa-se que alguns hábitos rastreados pelo CPV-A (como consumo de bebidas energéticas e a ingestão de água) apresentaram relação com a autoavaliação da voz por parte dos dubladores. A ingestão de bebidas energéticas tem particular relevância na amostra estudada, pois também esteve associada ao hábito de realizar desaquecimento vocal e a cinco sintomas do ITDV (voz grossa, rouquidão, tosse com secreção, secreção na garganta e cansaço ao falar). Ademais, este hábito também demonstrou associação com a autoavaliação ruim da audição (p-valor 0,004) e com a autopercepção de perda auditiva (p-valor 0,045). A cafeína, presente em altas dosagens nos energéticos, atua no Sistema Nervoso Central (SNC), impactando na cognição de forma a melhorar o tempo de atenção^(25-28)^ e impacta na produtividade dos indivíduos. Uma hipótese para este achado poderia estar relacionada às longas jornadas de trabalhos que os participantes indicaram (chegando a 60 horas semanais), culminando no uso destas bebidas para driblar o cansaço e cumprir as exaustivas jornadas de trabalho.

Destaca-se que o escore do ITDV foi associado com diversos sintomas vocais que estão presentes dentro do CPV-A, o que pode sugerir uma convergência entre as respostas dos participantes para tais sintomas e a pertinência de aplicação deste instrumento nesta população.

Dentre os participantes da pesquisa, 41,8% (n=23) *raramente* ou *nunca* conseguem se manter financeiramente e 80% (n=44) referem realizar outras atividades com uso da voz, muitas vezes em outras profissões (professor, ator de teatro, cantor, gravação de audiolivro e/ou voz original, locução, direção de dublagem, entre outras). Uma hipótese para este achado é que estas características nos participantes favorecem maiores riscos vocais e auditivos, pois pressupõe-se que o cenário a que estão submetidos propicia a necessidade de uma jornada laboral mais extensa, o que justificaria também o consumo de bebidas energéticas apontado acima.

Estudos sobre sintomas vocais em profissionais da voz são comuns, no entanto, recortes específicos sobre dubladores são mais raros. Estudo anterior^(29)^ apresenta que os sintomas mais relevantes para os dubladores após uma gravação foram: ressecamento na garganta, pigarros constantes, tensão no ombro e pescoço e rouquidão. Em nosso estudo, apesar da utilização de instrumentos diferentes do estudo citado anteriormente, estes sintomas vocais também foram relatados pelos participantes, mas apenas o pigarro apresentou respostas significativas, sendo referido pela maioria dos dubladores nas frequências *às vezes* e *sempre*. O pigarro também já foi referido em estudo com outras populações utilizando o CPV-P, como na comparação entre pastores evangélicos e grupo controle^(30)^. Por fim, obteve-se relação entre suspeita de DV e gênero, sendo maior nas mulheres. Este achado pode estar relacionado às condições anatômicas apresentadas por mulheres *cisgênero*, que se mostram favoráveis para o desenvolvimento de alterações vocais^(31-34)^.

Assim como todos os estudos científicos, este também possui algumas limitações, visto que não existe um instrumento validado para os dubladores e, portanto, utilizou-se o CPV-A com modificações em algumas perguntas para aumentar a especificidade para os a população do estudo; apesar destes também serem atores (o que justifica o uso do instrumento), durante seu trabalho em dublagem, estão sujeitos a demandas específicas. O ITDV é um instrumento validado construído para rastreio de distúrbio de voz em professores, porém, as questões referem-se a sintomas vocais comumente vivenciados por pessoas com distúrbios de voz e que não necessariamente têm relação com a docência; por isso, pode ser perfeitamente encaixado na amostra composta por atores. É importante ressaltar que neste estudo abordamos apenas a autopercepção destes sujeitos sobre sintomas vocais e auditivos. Apesar de representarem um item importante da avaliação multidimensional da voz, não é suficiente para uma avaliação detalhada da voz nestes profissionais. Em estudos futuros, é importante a investigação de julgamento perceptivo-auditivo e aspectos acústicos, além de exames audiológicos para confirmar ou refutar perda auditiva nesta população. Entretanto, os resultados obtidos chamam a atenção da necessidade de uma abordagem ampla na avaliação fonoaudiológica de dubladores, ressaltando os aspectos auditivos e vocais, com vistas à promoção da saúde destes trabalhadores.

## CONCLUSÃO

O sintoma vocal mais frequente para os participantes foi o pigarro, seguido pela rouquidão. A suspeita de distúrbio de voz atingiu menos participantes do que a autopercepção de perda auditiva. Houve associação entre o escore do ITDV e sintomas vocais e também com a audição. Apenas o sintoma pigarro apresentou associação significativa com o ITDV e o QPAAR. Considerando esse sintoma, os participantes que apresentaram ITDV negativo referiram menos perda auditiva autorreferida do que os dubladores com ITDV positivo.
